# Acute ocular toxoplasmosis presenting as a retinal angiomatous lesion

**DOI:** 10.5935/0004-2749.2023-0053

**Published:** 2023

**Authors:** Leticia Alcântara Pedroso, Flavia Veiga Costa, Amanda Gomes e Silva, Juliana Rocha de Mendonça da Silva, Ana Luiza Biancardi, André Luiz Land Curi

**Affiliations:** 1 Laboratory of Infectious Diseases in Ophthalmology, National Institute of Infectology, Fundação Oswaldo Cruz, Rio de Janeiro, RJ, Brazil; 2 Department of Ophthalmology, Universidade Federal do Rio de Janeiro, Rio de Janeiro, RJ, Brazil

To the Editor,

Ocular toxoplasmosis is the most common cause of posterior uveitis worldwide^(1)^. It typically presents as a unilateral, focal, necrotizing retinochoroidal lesion that appears as a whitish-yellow region with blurred margins, adjacent to or nearby a pigmented retinochoroidal scar, often associated with vitreous inflammation^(1)^. Although rare, atypical forms may occur.

We describe a case of a 57-year-old healthy woman complaining of blurred vision and ocular hyperemia in the right eye (OD) for 10 days. Her past ocular history was unremarkable. During the systems review, she reported being treated for primary syphilis with a single dose of benzathine penicillin G.

**Figure 1:**
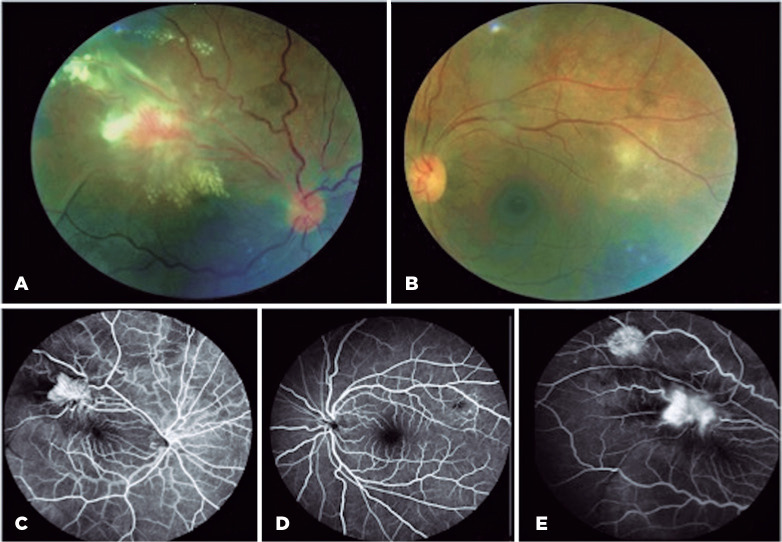
(A) Retinography of right eye (OD) showing two angiomatous exudative lesions in the superior temporal arcade with fibrovascular proliferation. (B) Retinography of left eye (OS) with small multifocal retinitis. (C, D, E) Fluorescein angiography; revealed hyperfluorescence during the early phases (C) followed by leakage suggestive of retinal neovascularization in the OD (E) and early hypofluorescent lesions due to retinal inflammation with delayed staining in the OS (D).

The ophthalmic examination revealed vision acuity (VA) of counting fingers in the OD and 20/20 in the left eye (OS). Anterior segment evaluation of both eyes showed granulomatous keratic precipitates and 2+ cells in the OD anterior chamber and 1+ cells in the OS. Fundus examination revealed vitritis with 2+ vitreous haze and two angiomatous exudative lesions in the superior temporal arcade with fibrovascular proliferation in the OD. The OS exhibited vitritis asso-ciated with small multifocal retinitis adjacent to the superior temporal arcade (Figure 1).

**Figure 2:**
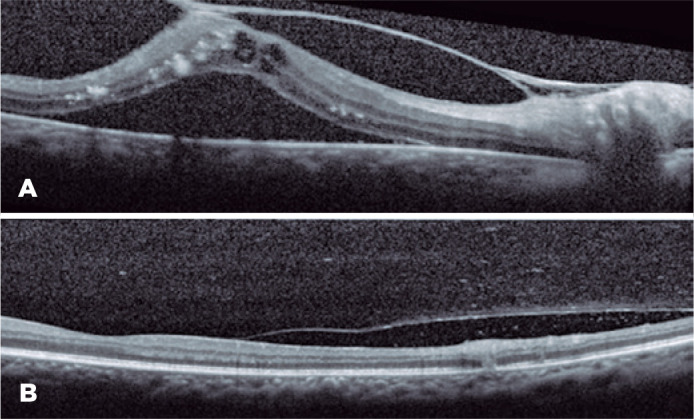
Optical coherence tomography (OCT) revealed tractional re-tinal detachment in the right eye (OD) with intraretinal fluid and hard exudates (A) and hyper-reflectivity of the inner retinal layers in the left eye (OS) (retinitis) with vitreous cells (B).

Ancillary tests were performed. Fluorescein angio-graphy showed hyperfluorescence during the early phases followed by leakage, suggesting retinal neovascularization in the OD and early hypofluorescent lesions due to retinal inflammation with delayed staining in the OS (Figure 1). Optical coherence tomography (OCT) revealed tractional retinal detachment in the OD with intraretinal fluid and hard exudates and hyper-reflectivity in the inner retinal layers in the OS (retinitis) with vitreous cells (Figure 2).

Serologic tests revealed positive immunoglobulin M (IgM) and immunoglobulin G (IgG) results for toxoplasmosis and negative for other infectious diseases.

Based on the serology result, a diagnosis of presumed atypical ocular toxoplasmosis was made, and treatment with sulfamethoxazole 800 mg and trimethoprim 160 mg twice daily was initiated for 30 days.

A fundus examination performed 15 days following the treatment revealed a flattening of the margins of the lesions in both eyes; VA improved to 20/200 in the OD and remained at 20/20 in the OS.

It is known that atypical forms of ocular toxoplasmosis most commonly occur in immunocompromised or elderly patients, sometimes presenting as large, multiple, and/or bilateral lesions. Patients who tested positive for IgM more frequently have a different phenotype of the disease, with mild ocular inflammation and macular involvement^(1)^.

Other atypical presentations described include punctate outer retinal toxoplasmosis, retinal vasculitis, pigmentary retinopathy mimicking retinitis pigmentosa, neuroretinitis, or other forms of optic neuropathy, and multifocal peripheral retinitis simulating acute retinal necrosis^(2)^.

The differential diagnosis of a retinal angiomatous lesion includes cat-scratch disease, retinal capillary hemangioma, and Coats disease.

Cat-scratch disease is a bacterial infection caused by Bartonella that is transmitted to humans through biting and scratching by cats. Ocular involvement in immunocompromised patients results in an infection of the vascular endothelium and may present as a subretinal mass associated with an abnormal vascular network^(3)^.

Retinal capillary hemangioma (RCH) is a benign retinal hamartoma that can be associated with von Hippel--Lindau disease. The tumor consisted of endothelial and stromal glial cells. It may manifest without any signs of uveitis, as a red or pink mass that protrudes into the vitreous cavity, which may be accompanied by a dilated and tortuous artery and a draining vein due to arteriovenous shunting^(4)^.

Coats disease is an idiopathic condition characterized by telangiectatic neovascular disease with intraretinal and subretinal exudation and fluid accumulation. It is unilateral and predominantly affects young males without causing ocular inflammation^(5)^.

We presented a case of a 57-year-old woman with bilateral granulomatous uveitis, an angiomatous retinal lesion, and retinitis with positive toxoplasmosis serology (IgM and IgG) with no evidence of any type of contact with cats. RCH, cat-scratch disease (CSD), and Coats disease can be excluded because of the absence of a well-defined pink mass with feeder vessels (seen in RCH) and the epidemiological characteristics of Coats disease and CSD.

Ophthalmologists should be aware of these unusual presentations of toxoplasmosis, including retinitis and angiomatous lesions, to avoid delays in diagnosis and treatment.
